# The association between serum adipokines levels with senile osteoporosis: a systematic review and meta-analysis

**DOI:** 10.3389/fendo.2023.1193181

**Published:** 2023-07-27

**Authors:** Jiangna Wang, Shiwei Liu, Yuxiang Zhao, Syed Shah Zaman Haider Naqvi, Ruixue Duan

**Affiliations:** ^1^ Department of Biochemistry and Molecular Biology, Basic Medical College, Shanxi Medical University, Taiyuan, China; ^2^ Department of Endocrinology, Shanxi Bethune Hospital, Shanxi Academy of Medical Sciences, Tongji Shanxi Hospital, Third Hospital of Shanxi Medical University, Taiyuan, China

**Keywords:** senile osteoporosis, leptin, adiponectin, chemerin, meta-analysis

## Abstract

**Objective:**

The clinical correlation between adipokines levels in the blood and the incidence of senile osteoporosis (SOP) has not been clearly studied. We conducted this meta-analysis to elucidate the relationship between three common adipokines levels (leptin, adiponectin, and chemerin) and the incidence of SOP.

**Methods:**

We searched databases such as CNKI, CBM, VIP, Wanfang, PubMed, Web of Science, Embase, and the Cochrane Library to collect articles published since the establishment of the database until July 30, 2022.

**Results:**

In total, 11 studies met the selection criteria. Our meta-analysis showed that serum leptin levels were significantly lower (mean difference [MD], -2.53, 95% CI: -3.96 to -1.10, *I^2^ = *96%), chemerin levels were significantly higher (MD, 30.06, 95% CI: 16.71 to 43.40, *I^2^ = *94%), and adiponectin levels were not significantly different (MD, -0.55, 95% CI: -2.26 to 1.17, *P* = 0.53, *I^2^ = *98%) in SOP patients compared with healthy older individuals with normal bone mineral density (BMD). In addition, correlation analysis showed that leptin levels were positively correlated with lumbar bone mineral density (LBMD) (r = 0.36) and femoral bone mineral density (FBMD) (r = 0.38), chemerin levels were negatively correlated with LBMD (r = -0.55) and FBMD (r = -0.48), and there were significant positive correlations between leptin and adiponectin levels and body mass index (BMI) (r = 0.91 and 0.97).

**Conclusions:**

The likelihood of having SOP was higher in older individuals with low levels of leptin and higher levels of chemerin. In addition, BMI was somewhat lower with low levels of leptin and adiponectin.

**Systematic review registration:**

https://www.crd.york.ac.uk/prospero/, identifier CRD42022356469.

## 1 Introduction

Osteoporosis is a group of systemic metabolic lesions caused by a significant decrease in bone production and bone mineral density (BMD), damage to the fine tissue structure of the skeleton, and fragility fractures. Its most important pathogenesis is the inactivation of osteoblasts and the activation of osteoclasts, which ultimately leads to a disruption of bone metabolism and a reduction in bone tissue (1). Osteoporosis is a disease that is extremely detrimental to bone health and can lead to an increased risk of fractures (2), with hip fractures being the most serious (3).

With the accelerated aging of the global population, the incidence of senile osteoporosis (SOP) has increased significantly. Unlike postmenopausal osteoporosis (PMOP), SOP is caused by advancing age and therefore does not only affect women but is also closely associated with men (4). In recent years, due to the increased prevalence of osteoporosis in the elderly population, the incidence of osteoporotic fractures has also increased, and the rate of death and disability has increased considerably. Therefore, as a common occult disease that seriously endangers the quality of life of the elderly, SOP has become one of the major public health problems worldwide.

Domestic and international studies have found that as we age, bone marrow stromal cells (BMSCs) differentiate into more adipocytes than osteoblasts, which leads to reduced bone formation and contributes to SOP (4). Adipokines are an emerging class of signaling factors produced mainly by adipocytes and are an important marker molecule of obesity. They are not only related to glucolipid metabolism (5) *in vivo*, but also have a connection with BMD (6, 7).

Furthermore, in addition to adipokines, adipose tissue and body mass index (BMI) play an extremely important role in osteoporosis. It was found that adipose tissue and BMI influence both bone density and bone turnover. Some studies found that adipose tissue has a certain mechanical protective effect on BMD, which can effectively prevent the occurrence of fractures after osteoporosis, and a significant association between BMD and BMI has been reported (r = 0.3 to 0.6) (8–10).

Several previous meta-analyses have examined the effects of adipokines levels on BMD and fracture risk in healthy men and women (7), the differences in adipokines between PMOP and healthy postmenopausal women (11), and the correlation of adipokines with BMD and BMI in PMOP patients (12). However, the clinical relevance of blood levels of adipokines in patients with SOP has not been clearly elaborated. Therefore, it is necessary to further explore the correlation between adipokines levels and SOP, BMI, and BMD to search for potential biomarkers to diagnose and treat SOP.

## 2 Materials and methods

We conducted a meta-analysis according to the Preferred Reporting Items for Systematic Reviews and Network Meta-Analyses (PRISMA) and Cochrane methods, and the completed PRISMA checklist is listed in **Table S1** and has been registered in the International Register of Prospective Systematic Reviews (PROSPERO) under CRD42022356469.

### 2.1 Search strategy

“Osteoporosis”, “senile osteoporosis”, “senile bone losses”, “SOP”, “adipokines”, “leptin”, “adiponectin”, and “chemerin” were used as our search terms in databases such as China National Knowledge Infrastructure (CNKI), CBM, VIP information database, Wanfang Data, PubMed, Web of Science, EMBASE, and Cochrane Library to search the articles since their establishment until July 30, 2022. This category was limited to human studies. The detailed search strategies are documented in **Table S2**.

### 2.2 Selection criteria

Two authors independently evaluated and selected relevant studies based on the following criteria: 1) study type: case-control studies and cohort studies; 2) study subjects: the elderly who meet the WHO diagnostic criteria for OP in the case group and healthy older individuals with normal BMD in the control group; 3) study index: data on leptin, adiponectin, or chemerin in SOP and control groups must be recorded completely; and 4) Newcastle Ottawa Scale (NOS) score ≥5. Exclusion criteria: 1) duplicate publications or overlapping study subjects; 2) the full text was not available; 3) relevant data were not provided in the original text or were incomplete and could not be obtained from the authors; 4) animal studies, case reports, editorials, reviews, expert opinion reports, and conference papers; and 5) study subjects suffered from diseases affecting bone metabolism or adipokines expression or had taken drugs affecting bone metabolism and lipid metabolism within 1 year.

### 2.3 Data extraction

From the included articles, two authors independently extracted the following information: 1) study characteristics, including first author, year of publication, and location; 2) basic characteristics of the individuals, including sample size, mean age, and mean BMI; and 3) details of biochemical examinations, including the source of the index and method of analysis. In addition, we extracted correlation coefficients (13) to further assess the relationship between different adipokines levels and BMI and BMD. When reporting Spearman correlation coefficients, we converted them to Pearson correlation coefficients according to the method described in previous studies (12, 14).

### 2.4 Quality assessment

Studies were evaluated for quality using the NOS score (15), with two researchers scoring each study and reviewing the literature separately. The NOS score included study population selection, comparability between groups, and exposure evaluation. The total score of ≥7, 5-6, and ≤5 was considered high, moderate, and low methodological quality, respectively.

### 2.5 Results of interest

In this meta-analysis, we labeled the differences in leptin, adiponectin, and chemerin concentrations between individuals with SOP and healthy older individuals as the primary outcome and the relationships between the concentrations of adipokines and BMI, LBMD, and FBMD as secondary outcomes.

### 2.6 Statistical analysis

All statistical analyses were performed using RevMan 5.4 software. Since adipokines levels were continuous variables and all indicators were calculated using comparable data units, effect sizes were combined by calculating the mean difference (MD), standard deviation (SD), and corresponding 95% confidence interval (95% CI). Due to inevitable clinical heterogeneity between studies, the random-effects model was a more appropriate method to calculate summary effect measures. The statistical heterogeneity of individual outcome studies was assessed using the Cochrane Q test (16) and the *I* ^2^ statistic (17). Subgroup analysis was performed to explore clinical heterogeneity based on the adipokines method of analysis. Sensitivity analysis was used to assess the stability of the meta-analysis results. In addition, we first calculated the transformed values of the correlation coefficient values using the Fisher transformation method. Subsequently, we performed a meta-analysis to calculate a summary of Fisher Z values based on the generalized inverse variance model. Finally, the summary r-values were transformed from Fisher’s Z-values summary using accepted formulas. Because tests for publication bias were not reliable when <10 studies were included, we did not compare their publication bias by generating funnel plots. *P*-values <0.05 were considered statistically significant differences.

## 3 Results

### 3.1 Literature search

**Figure 1:**
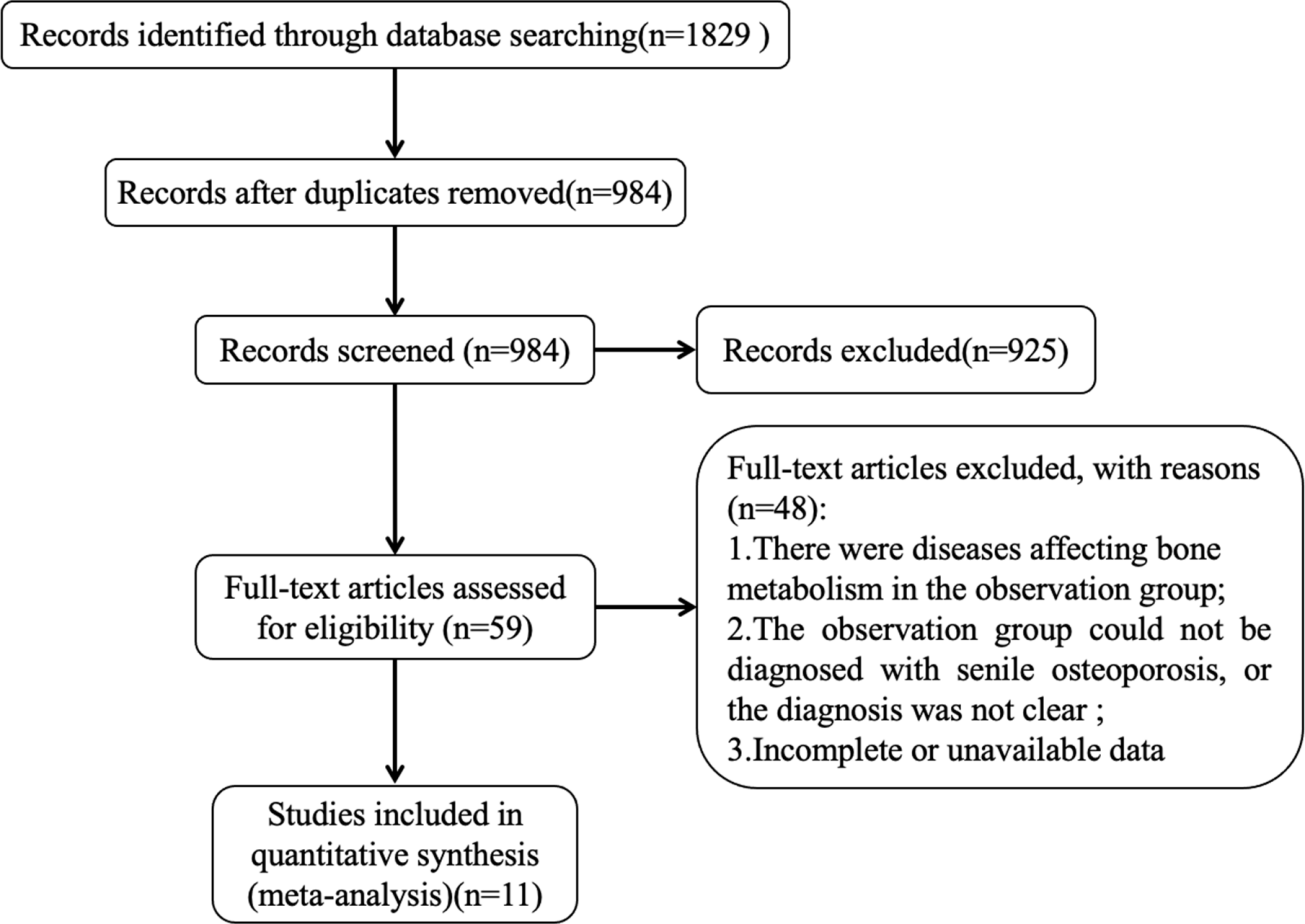
Flow diagram of literature screening.

After the initial searching, we found 1829 records, used ENDNOTE X9 and manual weight-checking methods to remove 845, then excluded 925 ineligible studies after carefully screening titles and abstracts, read the full text to remove 48 studies that did not meet the inclusion criteria, and finally identified 11 (18–28) studies were included for meta-analysis, with a total of 1337 study subjects. The literature screening process and results are shown in **Figure 1**.

### 3.2 Basic information and quality evaluation of included studies

Finally, a total of 11 studies were included in this meta-analysis, including 777 patients with SOP and 560 healthy older individuals with normal BMD. Of these studies, 10 studies (18–27) were published in China, and the remaining one was published in the United States (28). Nine (18–22, 24, 26–28) of these studies reported only one of the three common adipokines, and two studies (23, 25) reported multiple indicators of adipokines. And three studies (18, 19, 21) used radioimmunoassay (RIA) to measure serum adipokines levels, while the remaining eight (20, 22–28) used enzyme-linked immunosorbent assay (ELISA). The basic characteristics of the included studies are shown in **Table 1**. NOS was used to assess the methodological quality of the included studies. One study scored 5, four studies scored 6, four studies scored 7, and two studies scored 8. Overall, the overall methodological quality of the included studies was at a moderate to high level.

**Table 1:** Characteristics of the included studies (n = 11).

		Sample size (n)		Mean age (years)		Mean BMI (kg/m^2^)						
Study	Country	SOP	Control	SOP	Control	SOP	Control	Source	Indicators	Assay approach	r^a^	Score^b^
Shen,2006	China	88	70	75.36 ± 7.03	75.2 ± 6.27	24.29 ± 2.89	24.08 ± 13.31	Serum	LP	RIA	BMI	6
Ba,2011	China	25	22	68.32 ± 13.40	67.95 ± 14.16	NA	NA	Serum	LP	RIA	NA	6
Yan,2020	China	30	30	73.20 ± 6.84	74.33 ± 7.41	21.34 ± 3.45	21.04 ± 3.11	Serum	LP	ELISA	BMI	7
Liu,2009	China	86	50	72.92 ± 9.186	68.8 ± 10.055	23.975 ± 3.31	26.594 ± 2.48	Serum	LP	RIA	BMI, BMD	5
Chai,2012	China	89	50	70.26 ± 5.27	69.89 ± 5.21	NA	NA	Serum	LP	ELISA	BMD	7
Liang,2022	China	72	65	66.76 ± 5. 84	66. 29 ± 5. 47	25. 26 ± 3. 10	25. 93 ± 2.79	Serum	LP, ADP, CHEM	ELISA	BMI, BMD	8
Chai,2013	China	89	50	70. 26 ± 5. 27	69.89± 5. 21	NA	NA	Serum	ADP	ELISA	BMD	6
Liang,2019	China	70	70	71.53 ± 7.99	66.55 ± 6.12	22.99 ± 2.99	24.65 ± 2.12	Serum	LP, ADP	ELISA	BMI, BMD	7
Zhang,2013	China	33	31	79.1 ± 9. 5	74.7 ± 8.7	25. 72 ± 2. 07	26. 38 ± 1. 69	Serum	ADP	ELISA	BMD	7
Wang, 2016	China	84	82	85.85 ± 5.13	84.60 ± 5.00	NA	NA	Serum	ADP	ELISA	BMD	6
Jiang,2022	America	111	40	70.2 ± 5.7	69.1 ± 2.4	22.77 ± 2.75	23.63 ± 2.41	Serum	CHEM	ELISA	BMI.BMD	8

BMI, body mass index; SOP, senile osteoporosis; LP, leptin; ADP, adiponectin; CHEM, chemerin; ELISA, enzyme-linked immunosorbent assay; RIA, radioimmunoassay; NA, not available.

a Pearson’s or Spearman’s correlation coefficient.

b The methodological quality was determined using the Newcastle-Ottawa Scale (NOS).

### 3.3 Meta-analysis of Adipokines levels in all patients with SOP

**Figure 2:**
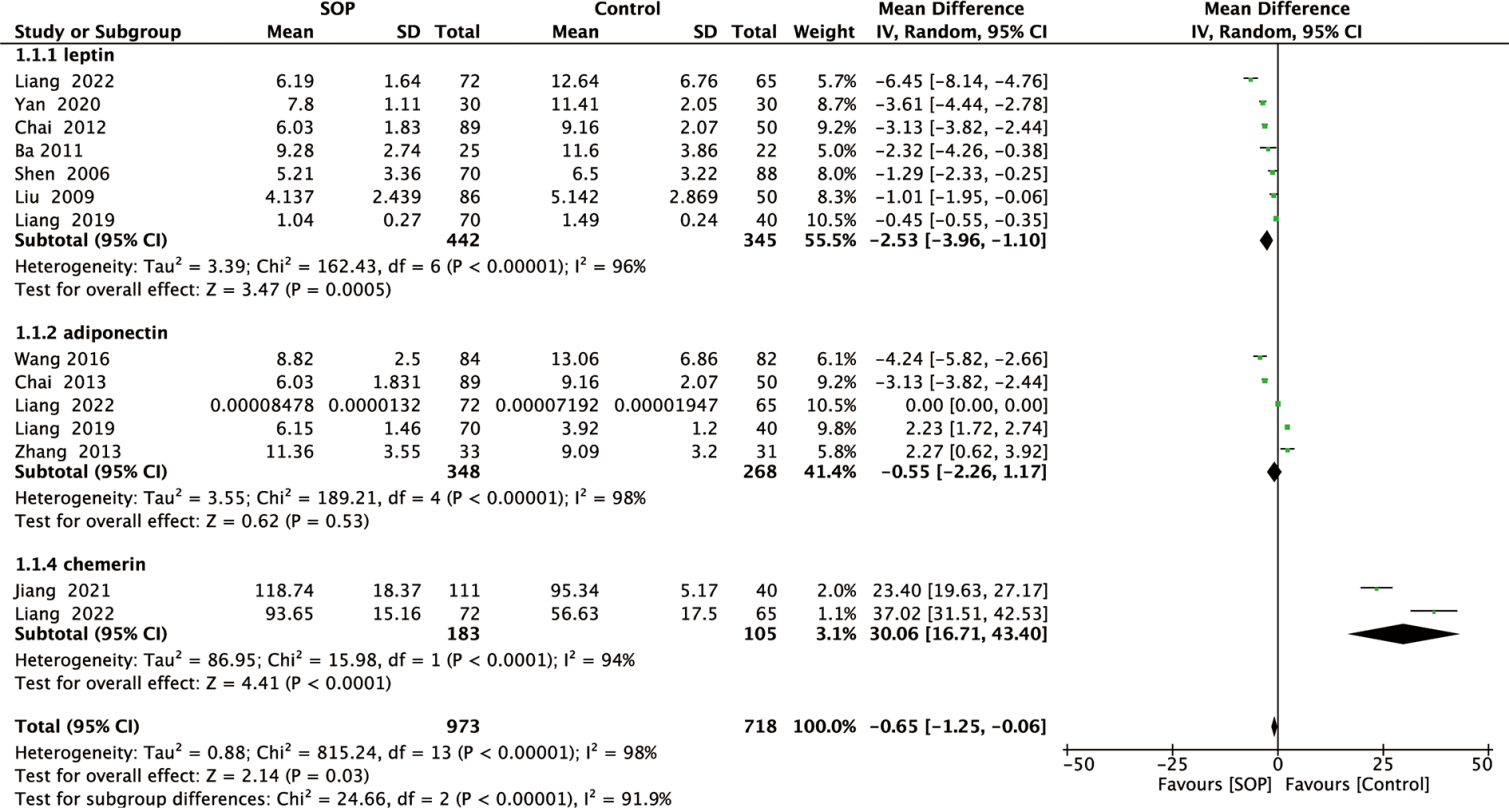
Forest plot of meta-analysis of differences in blood adipocytokines levels between individuals with senile osteoporosis and healthy patients with normal BMD.

**Figure 3:**
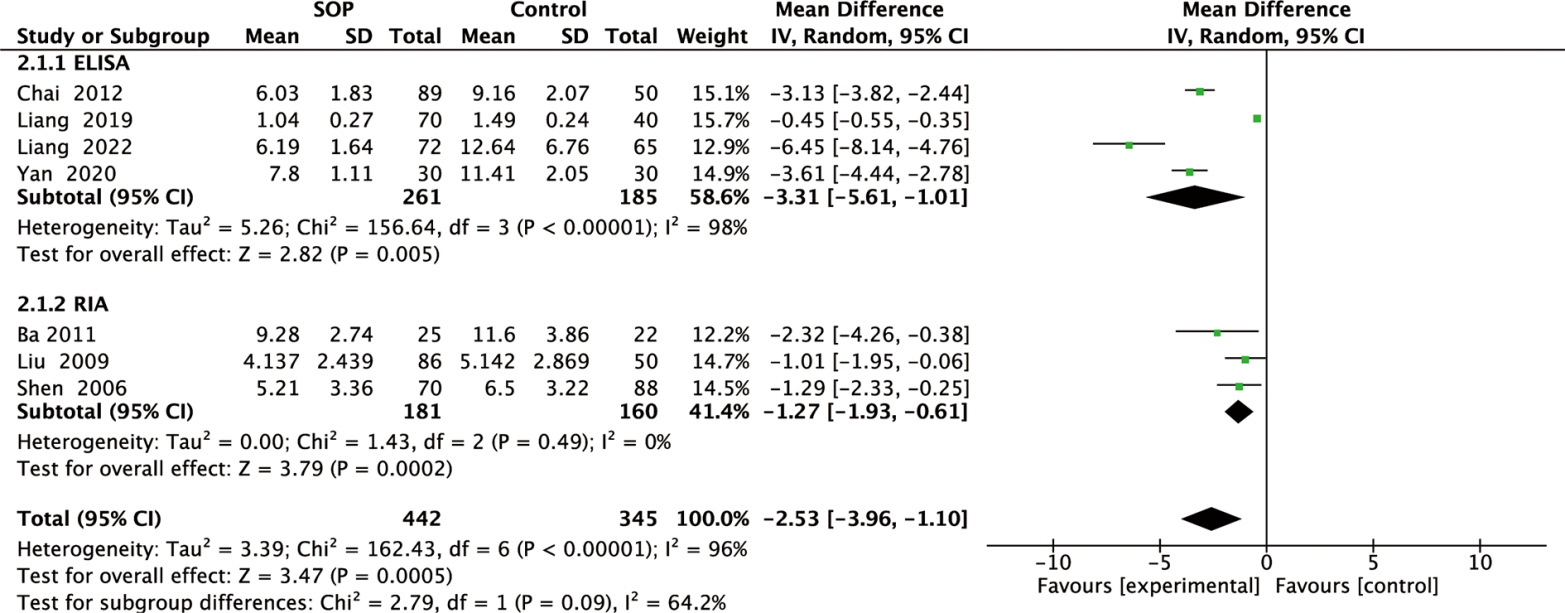
Forest plot of subgroup meta-analysis of differences in blood leptin levels between individuals with senile osteoporosis and healthy patients with normal BMD.

Among 11 studies included, 7 reported leptin levels in 442 SOP patients and 345 controls. Pooled results showed that leptin levels were significantly lower in SOP patients compared to controls (MD, -2.53, 95% CI: -3.96 to -1.10, *P* = 0.0005) (**Figure 2**). Significant heterogeneity was detected (*I^2^* = 96%, *P <*0.00001); therefore, we performed subgroup analyses based on leptin assay approaches to explore potential influencing factors. As shown in **Figure 3**, it was found that leptin levels in SOP patients in both the ELISA group (MD = -3.31, 95% CI: -5.61 to -1.01, *P* = 0.005) and the RIA group (MD = -1.27, 95% CI: -1.93 to -0.61, *P* = 0.0002) were statistically lower than in the control group, and there were no statistically significant differences between the two groups (*P* = 0.09), but statistical heterogeneity was not significantly reduced in the ELISA group (*I^2^ = *98%), while significantly reduced in the RIA group (*I^2 = ^* 0). And we performed a sensitivity analysis in which there was no significant change in the combined results after omitting one study at a time, indicating that it could confirm the robustness of the combined results (**Table S3.1**).

A total of five studies reported adiponectin levels in 348 SOP patients and 268 controls. As shown in **Figure 2**, adiponectin levels in SOP patients were not significant compared to controls (MD, -0.55, 95% CI: -2.26 to 1.17, *P* = 0.53). Although substantial statistical heterogeneity was detected (*I^2^ = *98%, *P <*0.00001), subgroup analysis was not performed because serum adiponectin levels were measured using ELISA in all studies. Sensitivity analysis has confirmed the robustness of the combined results (**Table S3.1**).

Finally, two studies reported chemerin levels in 183 SOP patients and 105 controls. As shown in **Figure 2**, serum chemerin levels were significantly higher in SOP patients than in controls (MD, 30.06, 95% CI: 16.71 to 43.40, *P <*0.0001). We detected significant statistical heterogeneity (*I^2^ = *94%, *P <*0.0001), but we did not perform subgroup analyses because serum chemerin levels were measured using ELISA in all studies. Moreover, sensitivity analysis has confirmed the robustness of the combined results (**Table S3.1**).

### 3.4 Meta-analysis of the relationship between adipokines levels and LBMD and FBMD

**Figure 4:**
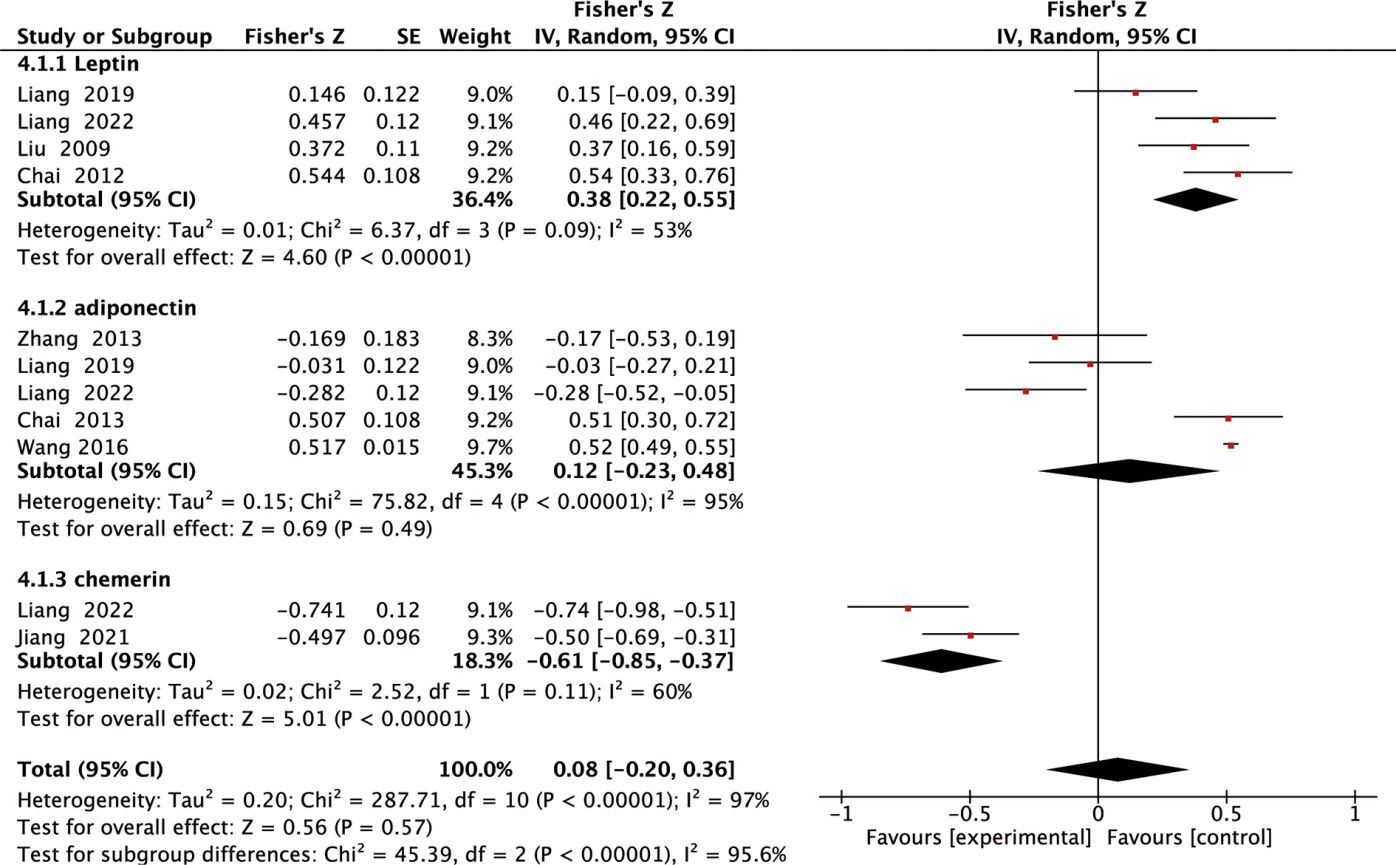
Forest plot of meta-analysis of the relationship between adipokines and LBMD.

**Figure 5:**
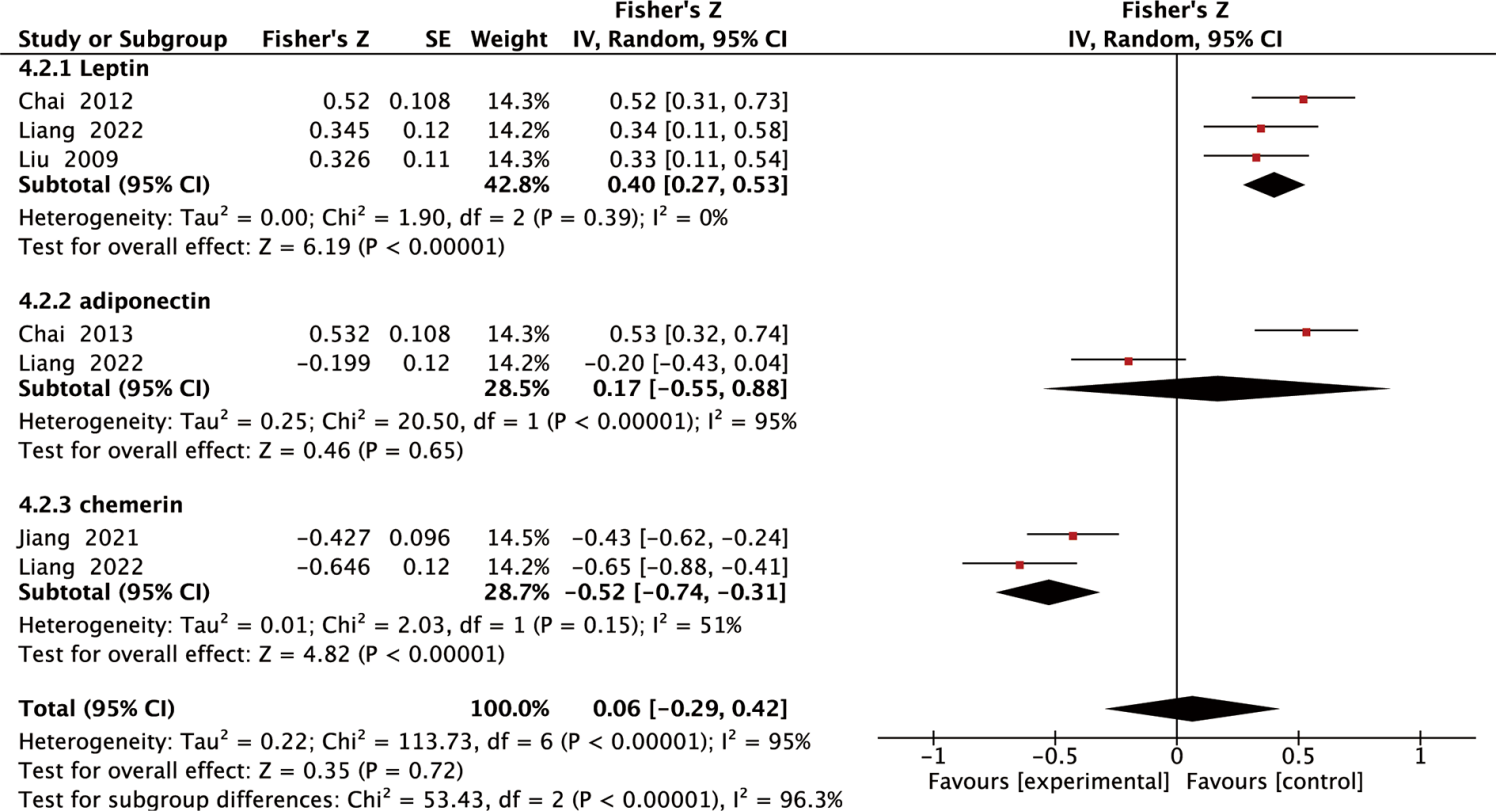
Forest plot of meta-analysis of the relationship between adipokines and FBMD.

Among the included studies, eight reported the relationship between adipokines levels and LBMD in individuals with SOP, five reported the relationship between adipokines levels and FBMD in individuals with SOP, and Pearson or Spearman correlation coefficients were calculated from the data. Using Fisher’s Z transform, we performed a pooled analysis of the correlations between adipokines levels and LBMD and FBMD in individuals with SOP. As shown in **Figure 4**, the Fisher’s Z values generated from the meta-analysis of the relationship between adipokines (leptin, adiponectin, and chemerin) levels and LBMD in patients with SOP were 0.38 (95% CI: 0.22 to 0.55, *P <*0.00001), 0.12 (95% CI: -0.23 to 0.48, *P* =0.49), and -0.61 (95% CI: -0.85 to -0.37, *P <*0.00001). Thus, the total r values for leptin, adiponectin, and chemerin were 0.36, 0.12, and -0.55, respectively. Analysis of the correlations showed that leptin levels were significantly and positively correlated with LBMD, and chemerin levels were significantly and negatively correlated with LBMD. Meanwhile, as shown in **Figure 5**, the Fisher Z values generated from the meta-analysis of the relationship between adipokines (leptin, adiponectin, and chemerin) levels and FBMD in SOP patients were 0.40 (95% CI: 0.27 to 0.53, *P <*0.00001), 0.17 (95% CI: -0.55 to 0.88, *P* = 0.65), and -0.52 (95% CI: -0.74 to -0.31, *P <*0.00001). Thus, the total r values for leptin, adiponectin, and chemerin were 0.38, 0.17, and -0.48, respectively. Analysis of the correlations showed that leptin levels were significantly and positively correlated with FBMD, and chemerin levels were significantly and negatively correlated with FBMD. Sensitivity analysis confirmed the robustness of the combined leptin and chemerin results (**Table S3.2**).

### 3.5 Meta-analysis of the relationship between Adipokines levels and BMI

**Figure 6:**
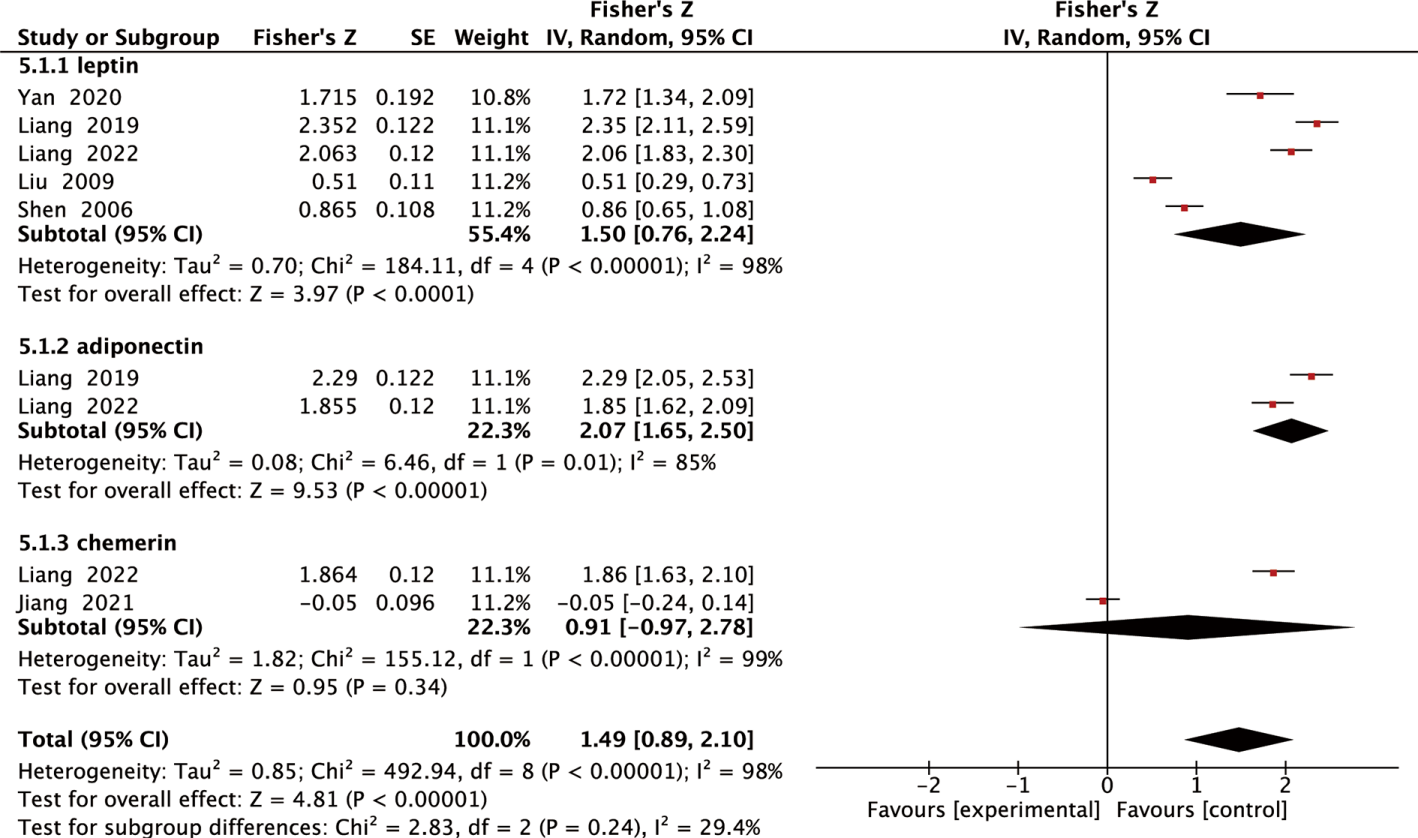
Forest plot of meta-analysis of the relationship between adipokines and BMI.

Six studies examined the relationship between leptin, adiponectin, and chemerin levels and BMI in individuals with SOP and reported or calculated Pearson or Spearman correlation coefficients. We performed a pooled analysis of the relationship between leptin, adiponectin, or chemerin levels and BMI in individuals with SOP. As shown in **Figure 6**, the meta-analysis generated pooled results for Fisher Z values of 1.50 (95% IC: 0.76 to 2.24, *P <*0.0001), 2.07 (95% IC: 1.65 to 2.50, *P <*0.00001) and 0.91 (95% IC: -0.97 to 2.78, *P* =0.34). Thus, the pooled r values for leptin, adiponectin, and chemerin were known to be 0.91, 0.97, and 0.72, respectively. The meta-analysis of correlation coefficients revealed significant positive correlations between leptin, adiponectin levels, and BMI, further confirmed by the sensitivity analysis results (**Table S3.3**).

### 3.6 Publication bias

We did not assess publication bias for leptin, adiponectin, and chemerin because the Cochrane Handbook of Interventional System Reviews (www.cochranehandbook.org) states that publication bias studies with funnel plots are not required when there are fewer than 10 studies.

## 4 Discussion

### 4.1 Comparison with existing research results

As three important adipokines, serum levels of leptin, adiponectin, and chemerin *in vivo* not only reflect the number and function of adipocytes but also play an important role in regulating bone metabolism. Among them, leptin affects bone metabolism in several ways. Leptin acts on the surface receptors of BMSCs, chondrocytes, osteoblasts, and osteoclasts, directly affecting bone metabolism (29); it also increases the expression of the insulin-like growth factor 1 receptor on the growth plate and condylar cartilage, indirectly regulating bone metabolism (30); in addition, it affects the secretion of bone marrow adipocytes, locally regulating bone metabolism (31). Turner et al. (32) showed that leptin-deficient mice had a heavier body weight, higher blood glucose levels, and more white abdominal adipose tissue, while shorter femur length, lower BMD, and thinner cortical thickness than normal mice. Consistent with these studies, leptin levels were found to be significantly lower in SOP patients than in controls in our meta-analysis and positively correlated with LBMD and FBMD. Several mechanistic studies have also found that leptin promotes the proliferation and differentiation of osteoblasts by activating the AKT (33) and the Janus kinase/signal transducer and activator of transcription (JAK/STAT) signaling pathways (34). In addition, leptin upregulates the OPG/RANKL ratio in BMSCs and inhibits the mitogen-activated protein kinases (MAPK) signaling pathway (35) to inhibit osteoclast differentiation. Thus, leptin can exert important effects on bone growth and metabolism through different pathways.

Some studies have shown that adiponectin has a dual effect on osteoporosis, on the one hand, it can bind to the adiponectin receptors R1 (AdipoR1) and AdipoR2 to positively regulate bone formation (36), on the other hand, it can negatively regulate bone metabolism by upregulating the RANKL/OPG ratio and downregulating the expression of the osteogenic transcription factor osterix (37). In contrast, our meta-analysis showed no significant difference between adiponectin levels in SOP patients and controls, which is consistent with the study by Barbour KE. et al. (38), where there was also no significant correlation between serum adiponectin levels and BMD and only a positive correlation with BMI. On the contrary, it is still found in some studies (39, 40) that occurrence of osteoporotic fractures in the elderly is significantly negatively correlated with serum adiponectin levels. In addition, a large number of mechanistic studies have also found that adiponectin can regulate the AMPK/FoxO3A signaling pathway (41), block the PI3K/AKT and Wnt/β-catenin signaling pathways (42), and adjust the differentiation direction of BMSCs. Although extensive studies, the exact role of adiponectin in bone metabolism remains controversial, and therefore, more studies are needed in the future to elucidate the complex effects of adiponectin on osteoporosis.

It was found that chemerin can regulate lipogenesis and osteogenesis in BMSCs, thereby dynamically regulating bone metabolism in the body. One study found that chemerin-deficient mice showed reduced bone mass and bone formation, whereas chemerin-overexpressing mice showed increased osteogenic differentiation and increased bone mass and bone formation (43). However, Jiang. et al. (28) found that prolonged exposure to chemerin enhanced osteoclast differentiation and maturation, thereby increasing the risk of fracture. As in the study, our meta-analysis showed that SOP patients had significantly higher chemerin levels than controls, and chemerin levels in SOP patients were negatively correlated with LBMD and FBMD.

In conclusion, the results of our meta-analysis suggest that older individuals with lower levels of leptin and higher levels of chemerin are more likely to have SOP. Interestingly, many studies showed a positive correlation between BMI and BMD, and low BMI is an important risk factor for osteoporosis (44, 45). Our results also showed that leptin levels were significantly and positively correlated with BMI and BMD in individuals with SOP, suggesting that it may play an important role in the process of BMI affecting the development of SOP, while how adipokines are involved in the process of BMI affecting osteoporosis needs further study due to the lack of relevant literature.

### 4.2 Strengths and limitations

Our study has unique advantages. First, this study is a cutting-edge analysis of studies designed to assess the clinical relevance of adipokines levels to SOP and explore other biomarkers that can predict and improve SOP. Second, our literature search and findings were comprehensive. Third, although some of the results showed heterogeneity, the quality of the included studies was at an intermediate to a high level of confidence, and we also performed a detailed subgroup analysis based on whether the assay approaches of different adipokines levels in blood influenced the result.

Of course, there are several limitations to consider. First, despite our best efforts to conduct subgroup analysis, the heterogeneity of each subgroup remained high, suggesting that there are still several unknown factors that may have contributed to study heterogeneity, such as geography, mean BMI, ethnicity, actual age, and nutritional status of older individuals. Second, our study included only case-control studies and lacked validated longitudinal cohort studies to infer a causal relationship between adipokines levels and the occurrence of SOP. Third, the correlation between adiponectin and SOP is different from some other studies, although some studies indicate that adiponectin predicted the incidence of osteoporotic fractures, our meta-analysis did not detect a significant correlation. Given the limited number of trials and small sample sizes for some outcomes, these findings may not be sufficient to ensure significant differences. Despite these limitations, our meta-analysis adds statistical power by pooling the results of individual studies, which means that the total number of subjects is large enough to support stronger conclusions.

### 4.3 Implications for practice and research

Currently, the prediction and diagnosis of osteoporosis and resulting fractures in the elderly are often based on imaging, but the use of imaging is limited to the analysis of only a single skeletal region, which is often not representative of the disease status of the entire skeleton in the elderly. Serum biomarkers may be more effective than imaging in predicting the status of the entire skeleton; moreover, BMD often does not reflect short-term changes in bone status during treatment, and complete reliance on BMD to predict treatment outcome and prognosis would be biased, whereas serum biomarkers can reflect bone metabolism in patients in the short term, which can improve the sensitivity and specificity of osteoporosis detection and monitoring in the continuous metabolism of bone tissue. In conclusion, the meta-analysis showed that the elderly with low leptin levels and high chemerin levels were more likely to have SOP, and BMD was positively correlated with leptin and negatively correlated with chemerin. However, the underlying mechanism of dysregulation of adipokines levels and whether correction of dysregulation of adipokines levels can be used to treat SOP and reduce the risk of osteoporotic fractures remains to be investigated.

## Data availability statement

The original contributions presented in the study are included in the article/**Supplementary Material**. Further inquiries can be directed to the corresponding author.

## Author contributions

JW and SL designed the project. JW and YZ collected and sorted the data. SL and RD reviewed the data. JW completed the first draft. SN revised the draft. SL, YZ, and RD reviewed the full text. and SL provided fund support. All authors contributed to the article and approved the submitted version.
